# Supplementary material for: Late Cenozoic unification of East and West Antarctica
Source: Nat Commun. 2018 Aug 9;9:3189. doi: 10.1038/s41467-018-05270-w (PMC6085322; doi:10.1038/s41467-018-05270-w)
Supplement: Supplementary file 1 — Supplementary Information [file 41467_2018_5270_MOESM1_ESM.pdf]

Supplementary Information

Late Cenozoic unification of East and West Antarctica

Granot and Dyment

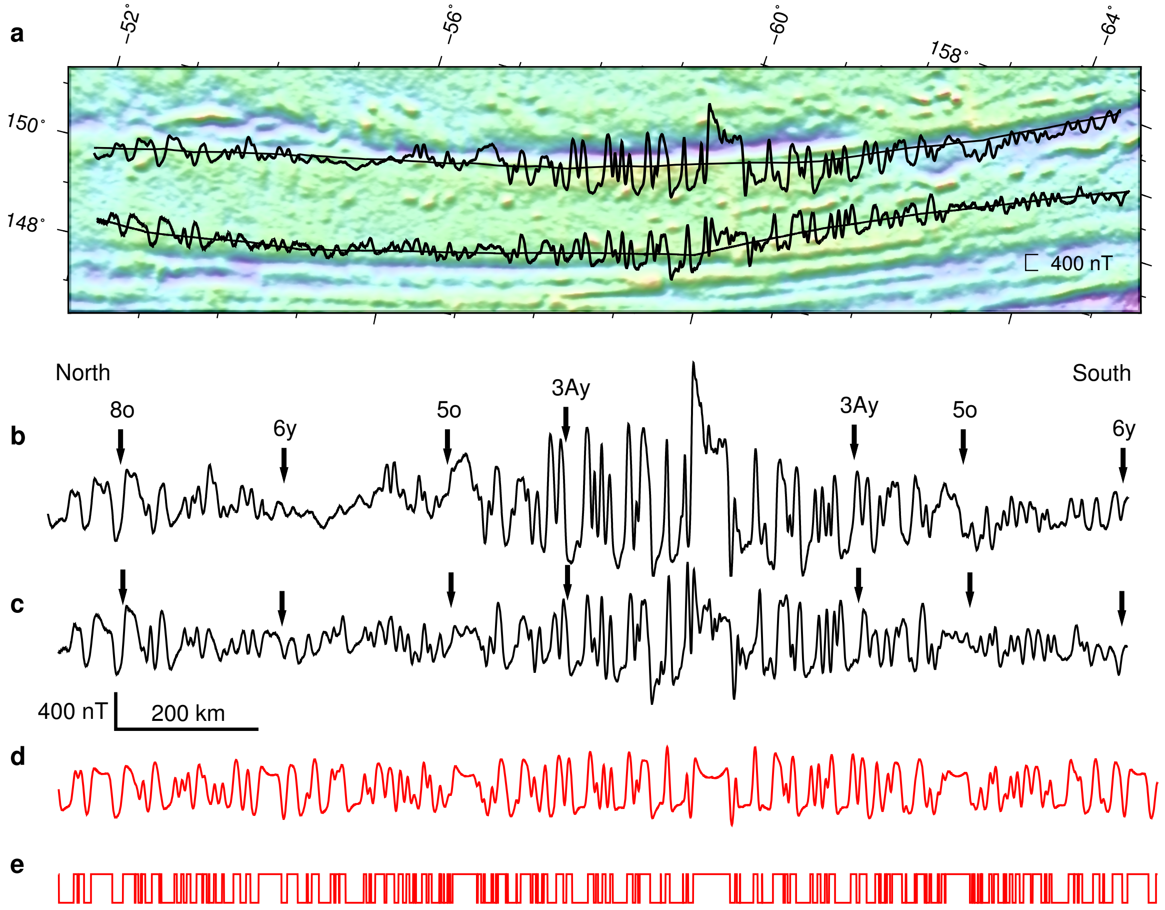

Supplementary Figure 1. Magnetic profiles collected along the Tasman corridor and forward model. (**a**) Total field magnetic anomalies plotted along the tracks acquired by the IB L'Astrolabe. (**b**) Eastern profile (TACT02 cruise). Locations of anomalies 3Ay, 5o, 6y and 8o are indicated with arrows. (**c**) Western Profile (TACT01 cruise). (**d**) Synthetic magnetic anomaly profile calculated based on magnetic block model shown in panel (**e**).

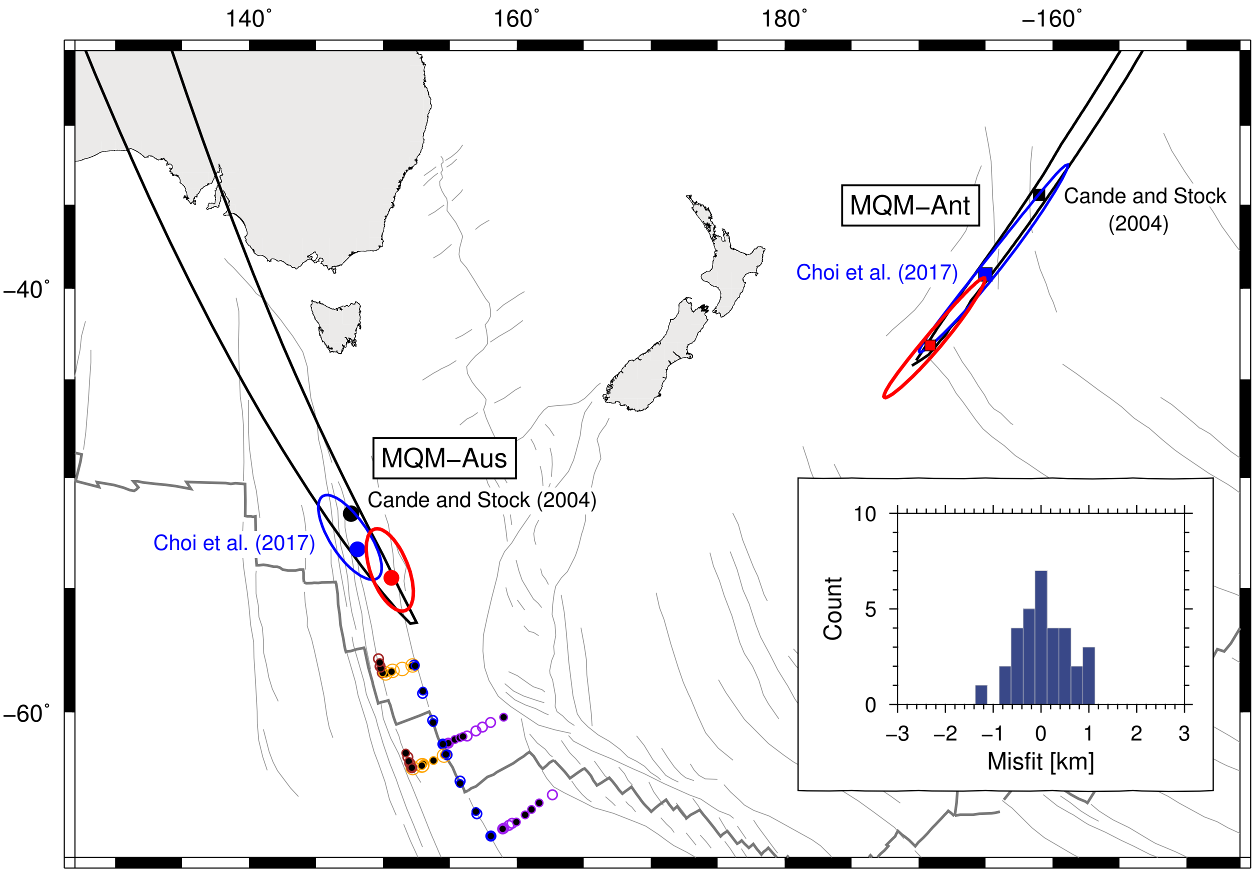

Supplementary Figure 2. Locations of the data used to constrain the motion of the Macquarie microplate (MQM) relative to Australia. The positions of the picks and fracture zones crossings (open symbols) of magnetic anomaly 3Ay are compared against the data from the conjugate plate (filled symbols) rotated over the Macquarie-Antarctica rotation. The resulting pole (red square) and its 95% confidence region (red ellipse, Table 1) are compared against previous kinematic solutions[1,2]. The Macquarie-Australia pole (red circle) and its 95% confidence region (red ellipse, Table 1), calculated by summing the Macquarie-Antarctica rotation parameters with the Australia-East Antarctica rotation parameters[1], are compared to previous kinematic solutions[1,2]. Inset shows the misfits of the reconstructed data used to constrain the Macquarie-Antarctica pole from the best-fit solution.

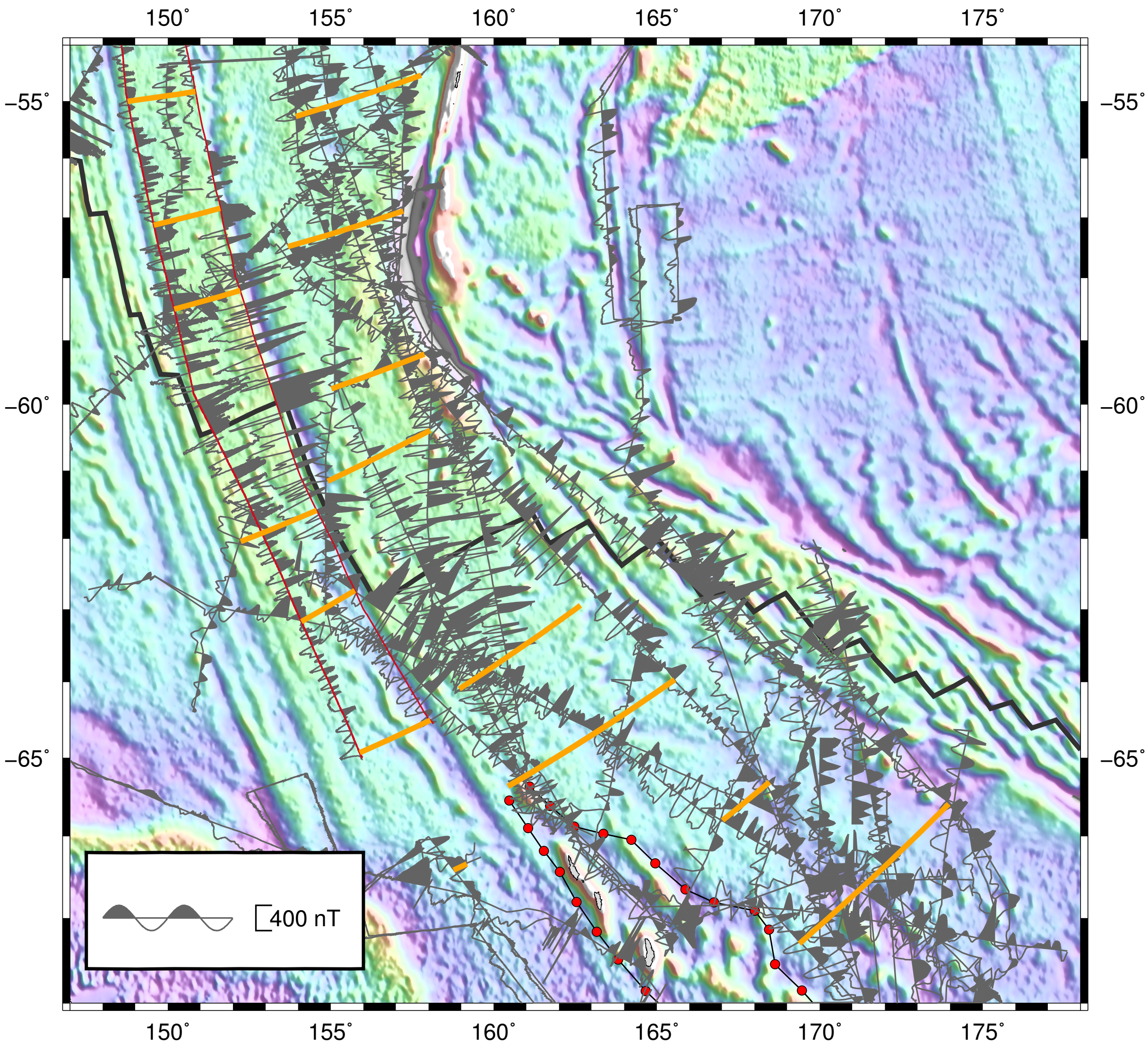

Supplementary Figure 3. Marine magnetic anomaly data used to locate the isochrons shown in Fig. 2. The archived data, obtained from the National Centers for Environmental Information (NCEI), are depicted by black tracks. Solid red lines marks TACT cruises. Grey shading indicates positive anomalies. Orange lines mark the locations of the mapped isochrons.

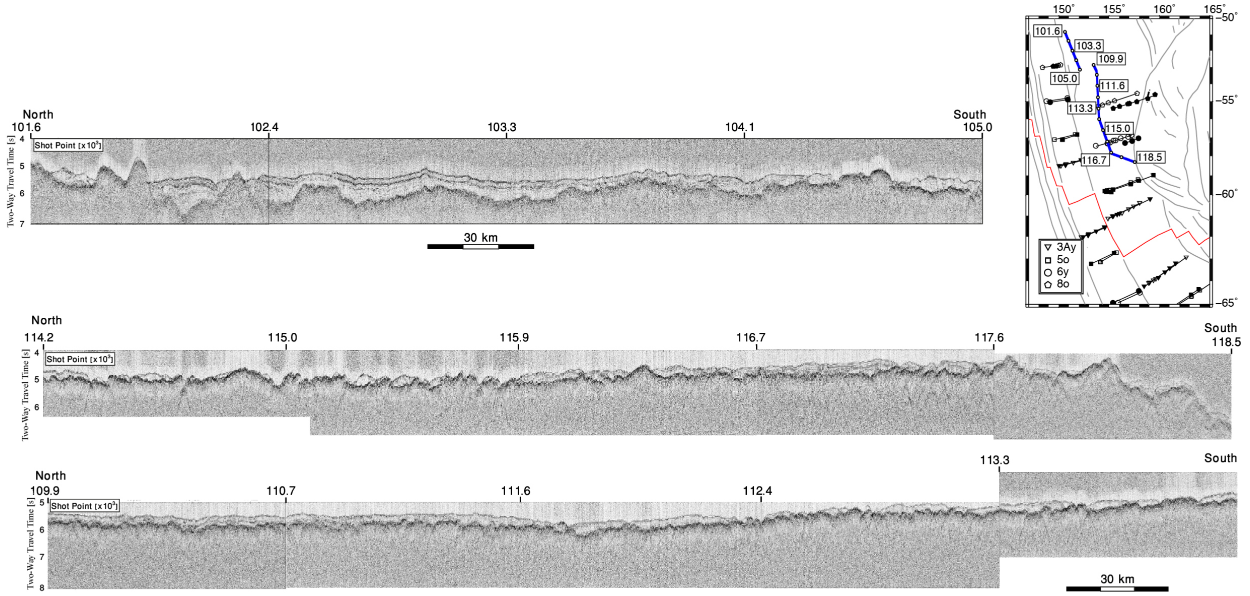

Supplementary Figure 4. Single-channel seismic profiles collected by RV Ewing cruise 9513. The profiles cross the Macquarie microplate within the Balleny corridor (see blue lines on inset map). Shot points are labeled for reference.

Supplementary References

1. Cande, S. C. & Stock, J. M. Pacific-Antarctic-Australia motion and the formation of the Macquarie Plate. *Geophys. J. Int.* **157**, 399-414 (2004).
2. Choi, H. *et al.* The kinematic evolution of the Macquarie Plate: a case study for the fragmentation of oceanic lithosphere. *Earth Planet. Sci. Lett.* **478**, 132-142 (2017).
